# Metabolic Response of *Sanghuangporus baumii* to Zn^2+^ Induction and Biosynthesis of a Key Pharmacological Component: Triterpenoid

**DOI:** 10.3390/microorganisms13051067

**Published:** 2025-05-03

**Authors:** Xinyu Tong, Ying Yu, Jin Huang, Ying Xu, Anxin Wang, Zengcai Liu, Li Zou

**Affiliations:** 1 College of Forestry, Northeast Forestry University, Harbin 150040, China; 18846783859@163.com (X.T.); yuying00@nefu.edu.cn (Y.Y.); xy3218935422@163.com (Y.X.); 17661291539@163.com (A.W.); 2 College of Chemistry, Chemical Engineering and Resource Utilization, Northeast Forestry University, Harbin 150040, China; huangjin@nefu.edu.cn

**Keywords:** Zn, *Sanghuangporus baumii*, triterpenoid, *HMGS* gene, heterologous biosynthesis, *Saccharomyces cerevisiae*

## Abstract

Triterpenoids derived from *Sanghuangporus baumii* exhibit potent antitumor activity, but their yields under natural conditions are relatively low due to their status as secondary metabolites. In this study, we investigated the effects of Zn²⁺ induction on the growth and triterpenoid biosynthesis of *S. baumii*. The results showed that 0.5 mM Zn²⁺ significantly enhanced the mycelial growth rate (0.43 ± 0.004 cm/d) and biomass (4.8 ± 0.024 g/L), representing increases of 8.71% and 16.95%, respectively, compared with the Zn0 group. This result was mainly caused by an increase in the soluble sugar content. Furthermore, 5 mM Zn²⁺ induced upregulation of genes in the mevalonate (MVA) pathway, thereby promoting triterpenoid accumulation by 167.86% compared with the Zn0 group. Transcriptome analysis identified *SbHMGS* as the key gene involved in triterpenoid biosynthesis under Zn²⁺ induction. Heterologous expression of *SbHMGS* in *Saccharomyces cerevisiae* confirmed its critical role in triterpenoid production. The triterpenoid (squalene) content of the engineered strain (*Sc*-*HMGS*) reached 0.88 mg/g under Zn²⁺ induction, which was 208.6% higher than in the non-induced control strain (*Sc*-NTC). These findings provide a foundation for optimizing the industrial fermentation condition of *S. baumii* and *S. cerevisiae* to enhance triterpenoid yields.

## 1. Introduction

*Sanghuangporus baumii* is a traditional Chinese medicinal mushroom known for its antitumor, immunomodulatory, and hypoglycemic effects. It is regarded as one of the most potent anticancer agents among mushrooms [1,2,3]. Triterpenoids, the primary bioactive components of *Sanghuangporus*, have attracted considerable attention due to their remarkable antitumor, antimicrobial, and antioxidant activities [4,5]. As the demand for triterpenoids continues to rise, there is a growing need for efficient and scalable production methods.

Chemical synthesis of triterpenoids faces challenges in large-scale applications due to inefficiency, high energy consumption, and adverse environmental impacts [6]. Currently, triterpenoids are predominantly extracted from the fruiting bodies or mycelia of *S. baumii* [5,7]. However, these secondary metabolites typically occur in low concentrations under natural conditions and are further constrained by climate, pests, and the cultivation scale, making it difficult to obtain a stable, high-yield supply. In a related study, Xu et al. demonstrated that adding metal ions to *Ganoderma lucidum* fermentation enhanced triterpenoid levels by 3.7-fold and led to the identification of several key genes involved in triterpenoid biosynthesis [8,9]. This finding highlights the utility of metal ion induction as an effective approach for promoting triterpenoid accumulation and uncovering crucial biosynthetic genes in macrofungi. Notably, heterologous synthesis of target compounds via the introduction of exogenous key genes into microbial hosts has become a prominent research focus [10]. Several triterpenoid compounds—such as ursolic acid [11], ganoderic acids [12], and betulinic acid [13]—as well as the triterpenoid precursor 2,3-oxidosqualene [14] have been produced heterologously in *Saccharomyces cerevisiae*, underscoring the feasibility of microbial engineering strategies. Together, these advances point to a promising route for the large-scale, controlled production of *S. baumii* triterpenoids.

Zn²⁺ is an essential trace element for the growth and metabolism of various organisms, as numerous enzymes and transcription factors require Zn²⁺ as a cofactor to function properly [15]. Previous studies have reported that Zn²⁺ induction can increase the polyphenol content in *Phellinus linteus* and promote the synthesis of *G. lucidum* polysaccharides [16,17]. However, there are currently no reports on Zn²⁺-induced triterpenoid biosynthesis in macrofungi. To address this gap, we investigated the effects of Zn²⁺ on the growth and triterpenoid production of *S. baumii* and identified the key gene involved in triterpenoid biosynthesis. Finally, the key gene was introduced into *S. cerevisiae* for heterologous expression. Our findings provide a foundation for large-scale, fermentation-based production of *S. baumii* triterpenoids.

## 2. Materials and Methods

### 2.1. Strain Culture and Zn^2+^ Treatment

For solid culture experiments, PDA medium was prepared with final Zn²⁺ concentrations of 0, 0.5, 1, 2, 5, and 10 mM by adding sterile ZnCl₂ stock solutions. These treatments are referred to as the Zn0, Zn0.5, Zn1, Zn2, Zn5, and Zn10 groups, respectively. Mycelial cakes of *S. baumii* (0.5 cm in diameter) were then placed onto the corresponding PDA plates and incubated at 25 °C. For the fermentation culture, 10 mycelial cakes were inoculated into 250 mL of PD medium and cultured at 25 °C and 180 rpm for 10 days to obtain seed liquid. The resulting seed culture was homogenized and transferred into fresh 250 mL PD medium at a 4% (*v*/*v*) inoculation rate, followed by incubation under the same conditions for 8 days. Subsequently, sterile ZnCl₂ stock solutions were added to match the final Zn²⁺ concentrations used in the solid culture experiments. These cultures were then induced for an additional 48 h at 25 °C. All treatments were carried out in triplicate.

### 2.2. Determination of Mycelial Growth Rate, Biomass, Triterpenoid Content, and Zn^2+^ Content

The mycelial growth rate was determined by measuring the average colony diameter in both the vertical and horizontal directions on PDA medium. Following fermentation, the broth was filtered, and the mycelia were washed three times with distilled water and then dried at 45 °C to a constant weight to determine the biomass. The triterpenoid content was assessed according to the method described by Liu et al. [18]. The Zn²⁺ concentration was quantified using inductively coupled plasma mass spectrometry (iCAP TQ, Thermo Fisher Scientific, Waltham, MA, USA), following standard protocols [19].

### 2.3. Transcriptome Sequencing of S. baumii

Samples exhibiting significant differences in growth rate and triterpenoid content were selected for transcriptome sequencing. Each sample (with three biological replicates) was sent to PANOMIX Biomedical Tech Co., Ltd. (Suzhou, China) for total RNA extraction and sequencing. Briefly, enriched and purified mRNA was fragmented into 200–300 bp fragments. Using these fragmented mRNA molecules as templates, cDNA was synthesized to create a cDNA library. After quality assessment of the library, high-throughput sequencing was performed on the Illumina platform. For transcriptome analysis, raw data were filtered to remove low-quality reads, and the remaining clean reads were assembled into unigenes. The expression levels of each unigene were calculated in FPKM. Differential expression analysis was performed using DESeq2 software, with |log₂(fold change)| > 1 and *p* < 0.05 as the criteria for defining differentially expressed genes (DEGs). GO and KEGG enrichment analyses of the DEGs were conducted simultaneously. Finally, nine DEGs were selected for qRT-PCR validation (primer sequences are provided in Table S2) to confirm the accuracy of the transcriptome data.

### 2.4. Quantification of Soluble Sugar Content

To evaluate the influence of Zn²⁺ treatment on *S. baumii* mycelia, the soluble sugar content was measured. Briefly, 0.1 g of mycelia was homogenized in 1 mL of distilled water, followed by incubation in a 95 °C water bath for 10 min. The homogenate was then centrifuged at 8000× *g* for 10 min. Subsequently, 0.1 mL of the resulting supernatant was mixed with 0.9 mL of distilled water, and the soluble sugar content was assessed using a KT-2-Y kit (Suzhou Comin, Suzhou, China) according to the manufacturer’s protocol.

### 2.5. Heterologous Expression of SbHMGS

To investigate the ability of *SbHMGS* in regulating triterpenoid biosynthesis, the *SbHMGS* gene was cloned into the pYES2-NTC vector via seamless cloning to construct pYES2-*HMGS*. This plasmid was then introduced into *S. cerevisiae* INVSc1 competent cells, yielding the recombinant strain *Sc*-*HMGS*. A control strain, *Sc*-NTC, was generated by transforming INVSc1 cells with the empty pYES2-NTC vector. Both the *Sc*-*HMGS* and *Sc*-NTC strains were inoculated into 50 mL of SD/-Ura medium for cell expansion at 28 °C and 180 rpm. When the optical density at 600 nm (OD₆₀₀) reached approximately 1.8, yeast cells were harvested by centrifugation, washed twice with sterile deionized water, and resuspended in 200 mL of SG/-Ura medium. Cultures were then incubated under the same conditions until the OD₆₀₀ again reached 1.8, at which point cells were induced for an additional 24 h to complete heterologous expression.

### 2.6. UPLC Analysis of Squalene in S. cerevisiae

Following heterologous expression, yeast cultures were centrifuged at 4000× *g* for 10 min, and the supernatant was discarded. The yeast cells were washed twice with deionized water and subsequently dried at 50 °C. For squalene extraction, 0.1 g of dried yeast cells was resuspended in 2 mL of a 10% KOH–75% ethanol solution, shaken thoroughly, and incubated in a boiling water bath for 15 min. After cooling, 2 mL of *n*-hexane was added, and the mixture was shaken well before centrifugation at 10,000× *g* for 5 min. The upper *n*-hexane phase was collected, and the extraction was repeated three times. The combined extracts were evaporated in a 60 °C water bath, and the residue was dissolved in 1 mL of acetonitrile. The solution was then filtered through a 0.22 μm membrane before UPLC analysis.

UPLC analysis was performed on an Agilent 1290 liquid chromatography system using a C18 column (5C18-PAQ, 4.6 × 250 mm, COSMOSIL, Kyoto, Japan). The column temperature was maintained at 20 °C with a flow rate of 1 mL/min, and detection was carried out at a wavelength of 203 nm. The mobile phase consisted of 100% acetonitrile, and the total run time for separation was 15 min. The squalene content was quantified based on a previously established standard curve [20].

### 2.7. Induction Culture of S. cerevisiae

To further enhance the triterpenoid yield in yeast, we first determined the optimal Zn²⁺ concentration for promoting triterpenoid accumulation. Specifically, the Sc-NTC strains grown in SG/-Ura medium to OD₆₀₀ =1.8 were supplemented with Zn²⁺ at 0, 0.5, 1, 2, 5, or 10 mM and incubated at 28 °C and 180 rpm for 24 h. Squalene was then quantified via UPLC to determine the optimal Zn²⁺ induction concentration. Based on these preliminary tests, the optimal Zn²⁺ concentration was applied to both the *Sc*-*HMGS* and *Sc*-NTC strains under the same conditions to assess the effect of Zn²⁺ on yeast triterpenoid production. Parallel controls received an equivalent volume of sterile deionized water in place of Zn²⁺.

### 2.8. Data Analysis

All data represent the means of three independent measurements. Error bars indicate standard deviations (SDs). Statistical significance (*p* < 0.05) was determined using IBM SPSS Statistics 25. Pearson correlation analyses were performed in Origin 2022.

## 3. Results

### 3.1. Effect of Zn^2+^ on the Growth and Triterpenoid Content of S.baumii

#### 3.1.1. Effect of Zn^2+^ on the Growth of *S. baumii*

**Figure 1:**
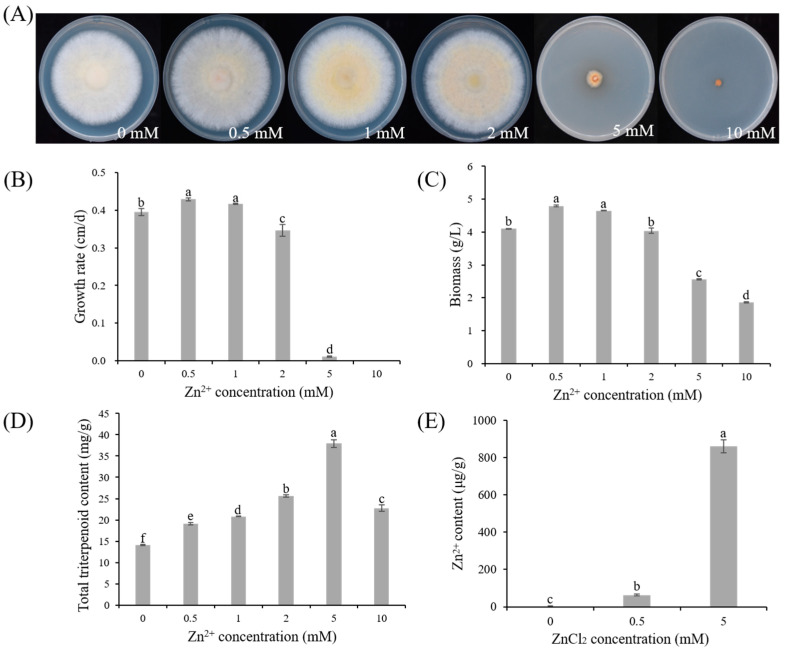
Effects of varying Zn^2+^ concentrations on *S. baumii* mycelia growth and triterpenoid production: (**A**) colony morphology; (**B**) mycelial growth rates; (**C**) biomass; (**D**) total triterpenoid content; and (**E**) intracellular Zn²⁺ content in *S. baumii* mycelia. Error bars represent the standard deviation (SD) of three replicates (*n* = 3). Letters indicate a significant difference (*p* < 0.05).

As shown in Figure 1A, increasing concentrations of Zn²⁺ caused the mycelia of *S. baumii* to transition in color from white to yellow and reduced the clarity of the colony edges. These changes became more pronounced at higher Zn²⁺ concentrations, indicating a dose-dependent influence on the colony morphology. Measurement of the mycelial growth rate revealed that as the Zn²⁺ concentration increased, the growth rate first rose and then declined. The Zn0.5 group exhibited the fastest growth rate (0.43 ± 0.004 cm/d), which was 8.71% higher than that of the Zn0 group, whereas mycelial growth was severely inhibited in the Zn5 group and absent in the Zn10 group (Figure 1B). A similar pattern was observed for the mycelial biomass, with the highest biomass in the Zn0.5 group (4.8 ± 0.02 g/L), being 16.95% higher than that in the Zn0 group (Figure 1C). Overall, these results indicate that low Zn²⁺ concentrations can promote *S. baumii* mycelial growth, whereas high Zn²⁺ concentrations inhibit it.

#### 3.1.2. Effect of Zn^2+^ on the Triterpenoid Content of *S. baumii*

In addition to influencing mycelial growth, Zn²⁺ also affected triterpenoid production in *S. baumii*. As shown in Figure 1D, Zn²⁺ concentrations ranging from 0.5 to 10 mM enhanced the triterpenoid content in *S. baumii* mycelia. Compared with the Zn0 group, the Zn0.5 group not only showed the highest mycelial growth rate but also exhibited a 34.83% increase in its triterpenoid content, reaching 19.11 ± 0.33 mg/g. The Zn5 group produced the greatest triterpenoid content, reaching 37.96 ± 0.92 mg/g, 167.86% higher than that of the Zn0 group. These results indicate that Zn²⁺ promotes triterpenoid biosynthesis in *S. baumii*, with 5 mM Zn²⁺ exerting the most significant effect.

#### 3.1.3. Zn^2+^ Content in *S. baumii* Mycelia under Zn^2+^ Treatment

Measurement of the Zn²⁺ levels in the *S. baumii* mycelia revealed a marked increase following Zn²⁺ supplementation. Compared with the Zn0 group (2.73 ± 1.29 μg/g), the Zn²⁺ content reached 63.21 ± 5.14 μg/g in the Zn0.5 group and 860.87 ± 34.18 μg/g in the Zn5 group (Figure 1E). These findings suggest that *S. baumii* mycelia can effectively absorb Zn²⁺, which in turn regulates both mycelial growth and triterpenoid biosynthesis.

### 3.2. Transcriptome Data Analysis and qRT-PCR Validation

#### 3.2.1. Overview of RNA-Seq Results

**Figure 2:**
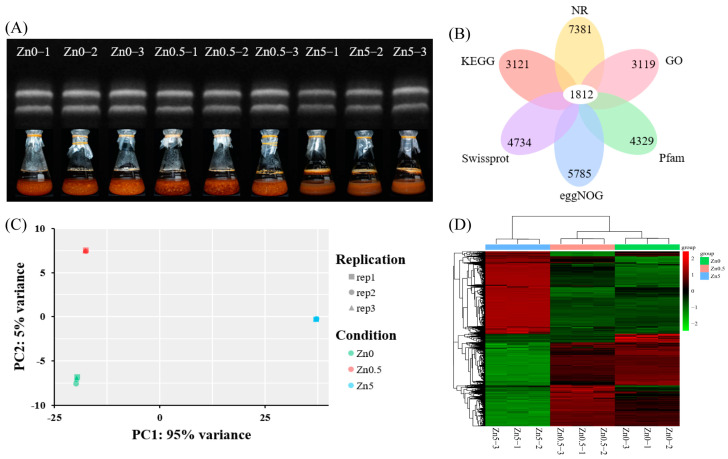
RNA-seq analysis of *S. baumii* under Zn^2+^ treatment. (**A**) RNA gel electrophoresis of nine samples. (**B**) Venn diagram showing functional annotation results in six public protein databases. (**C**) PCA of transcript profiles from Zn0, Zn0.5, and Zn5 groups. (**D**) Clustering heatmap illustrating expression patterns among samples (red = upregulation; green = downregulation).

The Zn0, Zn0.5, and Zn5 groups were selected for transcriptome sequencing, and the total RNA was extracted from the *S. baumii* mycelia (Figure 2A). A total of nine samples were sequenced, yielding 397,274,526 clean reads after filtering. The average clean read ratio was 98.56%, and the average Q30 score was 96.17% (Table S1). The assembled sequences were annotated in the NR, GO, KEGG, Pfam, eggNOG, and SwissProt databases. Specifically, 7381 sequences were annotated in NR, 3119 in GO, 3121 in KEGG, 4329 in Pfam, 5785 in eggNOG, and 4734 in SwissProt, representing 83.85%, 35.43%, 35.45%, 49.18%, 65.72%, and 53.78% of the total sequences, respectively. Among these, 1812 sequences were annotated in all databases, accounting for 20.58% of the total (Figure 2B). These results indicate that the RNA-Seq data quality was sufficiently high for further analyses.

The PCA results showed that the nine samples were separated into three distinct clusters, with each cluster containing three samples that were tightly grouped together, indicating high reproducibility within each treatment (Figure 2C). Differential gene expression analysis (Figure 2D) revealed relatively minor changes between the Zn0.5 and Zn0 groups, potentially involving genes related to mycelial growth. In contrast, significant gene expression changes were observed between the Zn5 and Zn0 groups, suggesting alterations in secondary metabolic pathways that likely contributed to the observed differences in triterpenoid production.

#### 3.2.2. Identification of DEGs and Analysis of Expression Pattern

**Figure 3:**
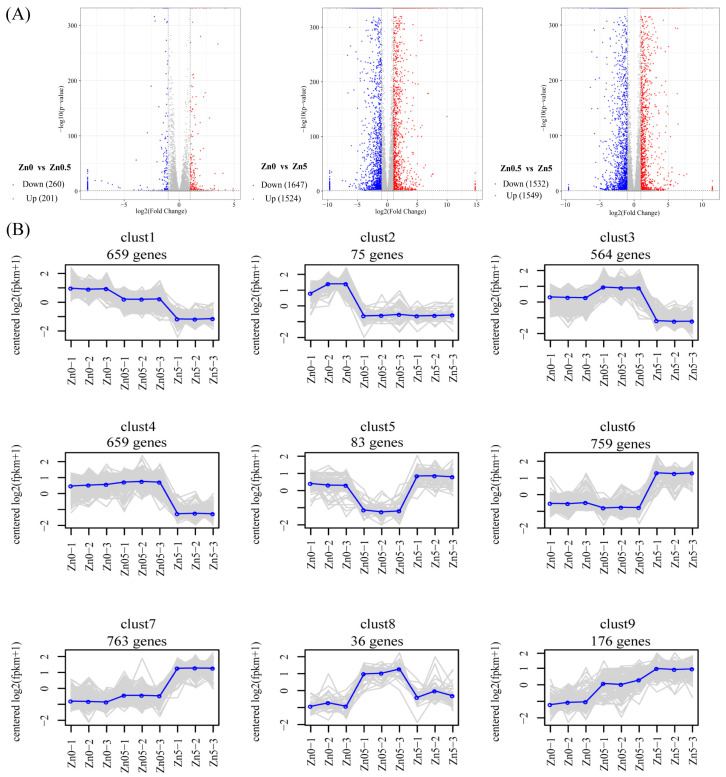
Analysis of DEGs in *S. baumii* under Zn^2+^ treatment. (**A**) Volcano plot of up- and downregulated DEGs from pairwise comparisons. (**B**) Expression patterns of DEGs s under Zn^2+^ treatment. Gray lines denote individual genes, and blue lines indicate the overall expression trend for each cluster. The number above each panel denotes the genes included in that expression pattern.

Between the Zn0 and Zn0.5 groups, 461 DEGs were identified, including 201 upregulated and 260 downregulated genes. Between the Zn0 and Zn5 groups, 3171 DEGs were identified, comprising 1524 upregulated and 1647 downregulated genes. Between the Zn0.5 and Zn5 groups, 3081 DEGs were identified, with 1549 upregulated and 1532 downregulated genes (Figure 3A). Additionally, 46 DEGs were unique to the Zn0 group versus the Zn0.5 group, 436 DEGs were unique to Zn0 versus Zn5, and 489 DEGs were unique to Zn0.5 versus Zn5 (Figure S1). These results underscore distinct transcriptional differences among the three treatments, especially between Zn5 and the other two groups.

On the basis of hierarchical clustering, DEGs were categorized into separate clusters according to their expression patterns (Figure 3B). Of the nine identified clusters, two (clust1 and clust2), comprising a total of 734 genes (659 in clust1 and 75 in clust2), showed higher expression in the Zn0 group. Three clusters (clust3, clust4, and clust8) with a combined total of 1259 genes (564, 659, and 36 genes, respectively) were highly expressed in the Zn0.5 group, aligning with the observed increase in mycelial growth. This suggests that these clusters may harbor genes associated with Zn²⁺-promoted mycelial proliferation. In contrast, four clusters (clust5, clust6, clust7, and clust9), totaling 1781 genes (83, 759, 763, and 176 genes, respectively), showed higher expression in the Zn5 group. The patterns in clust6, clust7, and clust9 corresponded to changes in the triterpenoid content, indicating that these gene clusters potentially govern Zn²⁺-enhanced triterpenoid biosynthesis.

#### 3.2.3. GO and KEGG Pathway Enrichment Analyses

**Figure 4:**
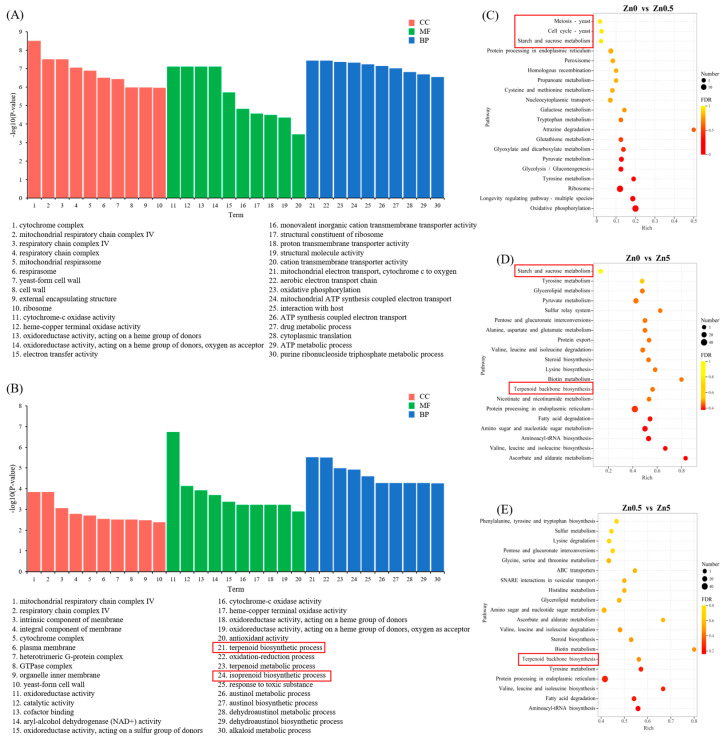
GO and KEGG enrichment analyses of DEGs under Zn^²⁺^ treatment. (**A**) GO classification of DEGs between Zn0.5 and Zn0 groups. (**B**) GO classification of DEGs between Zn5 and Zn0 groups. (**C**) KEGG enrichment analysis of DEGs between Zn0.5 and Zn0 groups. (**D**) KEGG enrichment analysis of DEGs between Zn5 and Zn0 groups. (**E**) KEGG enrichment analysis of DEGs between Zn5 and Zn0.5 groups.

To further investigate the effects of Zn²⁺ on *S. baumii*, GO and KEGG enrichment analyses were conducted on the DEGs identified among the Zn0, Zn0.5, and Zn5 groups. In the GO analysis, the top 10 terms with the lowest *p* values were selected from the cellular component (CC), molecular function (MF), and biological process (BP) categories. In the Zn0.5 versus Zn0 comparison, the DEGs were predominantly associated with cellular respiration, cation transmembrane transport, and energy metabolism (Figure 4A). In contrast, in the Zn5 versus Zn0 comparison, these DEGs were not only enriched in pathways related to the cellular structure and antioxidant activity but were also significantly enriched in the terpenoid biosynthetic process and isoprenoid biosynthetic process (Figure 4B). Within these latter categories, 13 and 14 genes were upregulated (Figure S2), accounting for 86.67% and 82.35% of the total DEGs in each pathway, respectively.

Similarly, the KEGG enrichment analysis (Figure 4C–E) indicated that, compared with the Zn0 group, DEGs in the Zn0.5 group were significantly enriched in the cell cycle and meiosis pathways. These DEGs, located predominantly in cluster 8, showed upregulated expression, implying an active phase of cytokinesis induced by 0.5 mM Zn²⁺, leading to enhanced mycelial growth. In accordance with the GO results, terpenoid backbone biosynthesis was significantly enriched in the Zn5 group—relative to both the Zn0 and Zn0.5 groups—and all corresponding DEGs were upregulated (Figure S3). These findings suggest that 5 mM Zn²⁺ may boost the triterpenoid content by upregulating genes involved in triterpenoid synthesis pathways.

#### 3.2.4. Soluble Sugar Content in *S. baumii* under Zn^2+^ Induction and DEGs Involved in Starch and Sucrose Metabolism

**Figure 5:**
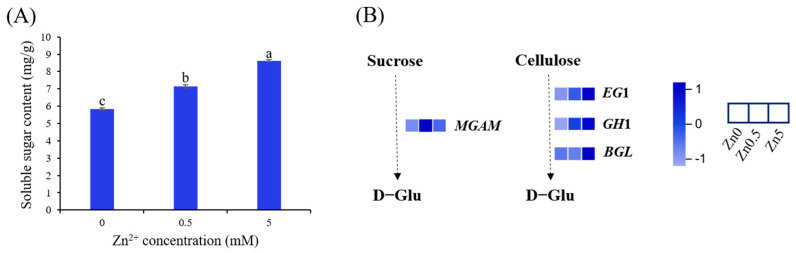
Changes in sugar metabolism of *S. baumii* mycelia induced by Zn^2+^. (**A**) Soluble sugar content in *S. baumii* under Zn^2+^ induction. (**B**) DEGs involved in starch and sucrose metabolism. Error bars represent the standard deviation (SD) of three replicates (*n* = 3). Letters indicate a significant difference (*p* < 0.05).

In the present study, the soluble sugar content was significantly higher in the Zn0.5 (7.15 mg/g) and Zn5 (8.63 mg/g) groups than in the Zn0 group (5.84 mg/g) (Figure 5A). Concurrently, KEGG enrichment analysis revealed that DEGs in both the Zn0.5 and Zn5 groups were enriched in the starch and sucrose metabolism pathway relative to the Zn0 group (Figure 4C,D and Figure 5B). For instance, in the Zn0.5 group, expression of the sucrose-degrading enzyme gene *MGAM* was markedly upregulated, whereas in the Zn5 group, the cellulose-degrading enzyme genes *EG*1, *GH*1, and *BGL* showed significant increases in expression. These findings suggest that Zn²⁺ elevates the soluble sugar content in *S. baumii* by regulating genes associated with carbohydrate metabolism. Although different Zn²⁺ concentrations may activate distinct response mechanisms, they ultimately converge on promoting the production of simple sugars, such as D-glucose.

#### 3.2.5. DEGs Involved in Terpenoid Backbone Biosynthesis

**Figure 6:**
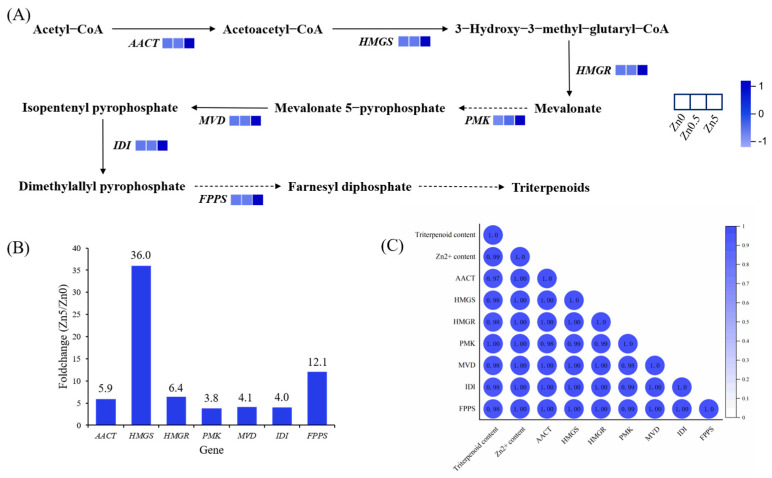
DEGs involved in terpenoid backbone biosynthesis. (**A**) Proposed pathways for triterpenoid biosynthesis in *S. baumii*. (**B**) Fold change of each MVA pathway gene in Zn5 vs. Zn0, where the numbers above the bars indicate the fold change. (**C**) Correlation analysis among the triterpenoid content, Zn^2+^ content, and gene expression level.

A schematic of the triterpenoid biosynthesis pathway in *S. baumii* is shown in Figure 6A. Seven genes in the MVA pathway exhibited progressively higher expression levels with increasing Zn²⁺ concentrations, and these genes were localized in clusters 6 and 7, consistent with the transcriptome-based gene expression pattern analysis. By comparing the fold change in expression between the Zn5 and Zn0 groups, the *HMGS* gene of *S.baumii* (*SbHMGS*) showed the greatest increase at 36-fold (Figure 6B). Correlation analyses among the Zn²⁺ content, triterpenoid content, and gene expression levels revealed a correlation coefficient of 0.98 between *SbHMGS* expression and the triterpenoid content and a perfect correlation of 1.0 between *SbHMGS* expression and the Zn²⁺ content (Figure 6C). These results indicate that *SbHMGS* plays a pivotal role in Zn²⁺-induced triterpenoid biosynthesis in *S. baumii*, warranting its selection as a key gene for subsequent experiments.

#### 3.2.6. qRT-PCR Validation

Using *α-tubulin* as the internal reference, nine genes involved in triterpenoid and sugar metabolic pathways were selected for qRT-PCR to validate the RNA-Seq data (primer sequences are listed in Table S2). The qRT-PCR expression profiles closely matched those obtained from RNA-Seq, confirming the reliability of the transcriptomic dataset (Figure S4).

### 3.3. Heterologous Expression of SbHMGS in S. cerevisiae

**Figure 7:**
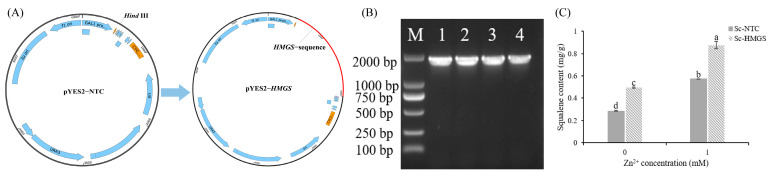
Heterologous expression of *SbHMGS* in *S. cerevisiae*. (**A**) Schematic of the pYES2-*HMGS* vector construction. (**B**) PCR product gel electrophoresis of four positive single colonies. (**C**) Squalene content in *Sc*-NTC and *Sc*-*HMGS* under Zn^2+^ induction. Error bars represent the standard deviation (SD) of three replicates (*n* = 3). Letters indicate a significant difference (*p* < 0.05).

To enhance squalene production, the key gene *SbHMGS* was introduced into *S. cerevisiae*. PCR and subsequent sequencing confirmed the successful construction of the engineered strain (*Sc-HMGS*; Figure 7A,B). Preliminary experiments determined that 1 mM Zn²⁺ yielded the highest squalene production in *S. cerevisiae*, reaching 0.57 mg/g (Figure S5). Subsequent fermentation experiments were conducted with *Sc-HMGS* and *Sc*-NTC, both with and without Zn²⁺ induction. In the absence of Zn²⁺, *Sc-HMGS* displayed a significantly higher squalene content than *Sc*-NTC. Under 1 mM Zn²⁺ induction, the squalene content of *Sc-HMGS* increased further, reaching 0.88 mg/g, 208.6% higher than that of untreated *Sc*-NTC (Figure 7C). These findings indicate that *SbHMGS* substantially boosts squalene accumulation in *S. cerevisiae*, and Zn²⁺ induction can further amplify this effect.

## 4. Discussion

Metal ions play critical roles in the growth, morphology, and physiology of macrofungi. Specifically, Zn²⁺ modulates enzyme activity and metabolic pathways. In this study, a low Zn²⁺ concentration (0.5 mM) promoted mycelial growth and biomass accumulation in *S. baumii*, whereas a high Zn²⁺ concentration (5 mM) inhibited these processes, consistent with previous findings [21]. Sugars function not only as structural components and energy sources but also as signaling molecules throughout an organism’s life cycle. Accordingly, the ability to sense and regulate sugar levels is essential for survival. Based on KEGG enrichment analyses and measurements of the soluble sugar content, we propose that under 0.5 mM Zn²⁺ induction, upregulation of genes associated with the cell cycle and meiosis (Figure 4C) accelerates mycelial division, resulting in enhanced growth and biomass. Concomitantly, Zn²⁺ exposure upregulated the sucrose hydrolase gene *MGAM* within the starch and sucrose pathway, thereby elevating intracellular glucose and promoting cell growth (Figure 5A). At 5 mM Zn²⁺, elevated intracellular Zn²⁺ likely causes cytotoxic effects and hampers mycelial expansion. In addition, the osmotic stress generated by elevated Zn²⁺ levels upregulated the cellulose-degrading enzymes *EG*1, *GH*1, and *BGL*. The resulting cellulose-to-glucose conversion (Figure 5B) increased intracellular glucose, which acted as an osmoprotectant, preserving cell viability [22]. This mechanism explains the observed rise in the soluble sugar content under 5 mM Zn²⁺ treatment.

Abiotic stress can significantly influence the production of secondary metabolites in mushrooms [23]. As shown in Figure 1D, the triterpenoid content in *S. baumii* reached its maximum at 5 mM Zn²⁺. Moreover, RNA-seq analysis showed that all DEGs in the MVA pathway were upregulated in the Zn5 group, supporting the conclusion that Zn²⁺ promotes triterpenoid accumulation in *S. baumii* by activating MVA pathway transcription. Within the MVA pathway, *HMGS* is recognized as a key enzyme in the irreversible reactions of terpenoid biosynthesis, and its expression can be strongly induced by exogenous factors [24,25,26,27]. Our transcriptomic data identified *SbHMGS* as the most prominently upregulated gene within the MVA pathway, with a positive correlation between its expression level and triterpenoid content, indicating that *SbHMGS* may serve as a principal target for Zn²⁺ induction. Because squalene is a vital precursor in triterpenoid biosynthesis, its intracellular concentration often limits the production of triterpenoids in *S. cerevisiae* [28,29]. In this study, heterologous expression of *SbHMGS* in *S. cerevisiae*, combined with 1 mM Zn²⁺ induction, yielded a squalene content of 0.88 mg/g, representing a 208.6% increase compared with the *Sc*-NTC strain. This highlights the feasibility of producing *S. baumii* triterpenes in *S. cerevisiae* through heterologous biosynthesis.

Beyond *S. baumii*, Zn²⁺ exerts comparable regulatory effects in a variety of species. For example, for mushrooms, supplementing growth substrates with low levels of Zn²⁺ significantly increased the yield of *Pleurotus ostreatus* [30]. For crops, foliar agronomic biofortification with low levels of Zn²⁺ enhanced lettuce yields, nutraceutical quality, antioxidant capacity, and leaf Zn accumulation [31]. These findings indicate that Zn²⁺ supplementation can act as a bio-fertilizer, promoting crop and mushroom growth. Conversely, Zn²⁺ also functions as an antimicrobial, inhibiting fungi, bacteria, and viruses [32,33]. Fungicides containing high concentrations of Zn²⁺ effectively suppressed the sporocarp formation and spore release of *Phytophthora capsici*, thereby reducing its infectivity on pepper leaves [34]. Collectively, these reports demonstrate that Zn²⁺ exerts concentration-dependent effects on biochemical processes, growth, and elemental compositions, ranging from beneficial to toxic. Determining species-specific optimal Zn²⁺ dosages is thus critical for maximizing desired outcomes while avoiding toxicity.

## 5. Conclusions

In this study, we demonstrated that the Zn²⁺ concentration substantially influences both *S. baumii* mycelial growth and triterpenoid accumulation. At 0.5 mM, Zn²⁺ promoted soluble sugar accumulation and stimulated cell division, thereby enhancing mycelial growth and biomass. Conversely, 5 mM Zn²⁺ disrupted the cellular osmotic balance and suppressed mycelial proliferation. Elevated Zn²⁺ levels also modulated the metabolic landscape of *S. baumii*, upregulating genes in the MVA pathway and leading to significant triterpenoid accumulation. Among these genes, *SbHMGS* played a pivotal role in triterpenoid biosynthesis. Heterologous expression of *SbHMGS* in *S. cerevisiae*, accompanied by 1 mM Zn²⁺ induction, markedly increased the squalene content. Collectively, these findings fill a critical gap in our understanding of Zn²⁺-induced responses in macrofungi and establish a yeast chassis with high squalene production, enabling the heterologous biosynthesis of *S. baumii* triterpenoids.

## Data Availability

All data that support the findings reported in this study are available from the corresponding authors and are shown in the supplemental data, which are an integral part of the article.

## Supplementary Materials

The following supporting information can be downloaded at https://www.mdpi.com/article/10.3390/microorganisms13051067/s1. Figure S1: Venn diagram of DEGs in diverse comparison groups. Figure S2: The percentage of upregulated DEGs to total DEGs in GO analysis comparing Zn5 and Zn0 groups. Figure S3: KEGG enrichment analysis of up- and downregulated genes in DEGs from diverse comparison groups. Figure S4: qRT-PCR validation of nine selected DEGs identified by RNA-Seq. Figure S5: Squalene content of *Sc*-NTC induced by different Zn^2+^ concentrations. Table S1: transcriptome data statistics. Table S2: Information about primers used in the present study.
