# Supplementary material for: Metabolic Response of Sanghuangporus baumii to Zn2+ Induction and Biosynthesis of a Key Pharmacological Component: Triterpenoid
Source: Microorganisms. 2025 May 3;13(5):1067. doi: 10.3390/microorganisms13051067 (PMC12113684; doi:10.3390/microorganisms13051067)
Supplement: Supplementary file 1 [file microorganisms-13-01067-s001.zip › microorganisms-3543980-supplementary.pdf]

Supplementary materials

Figures:

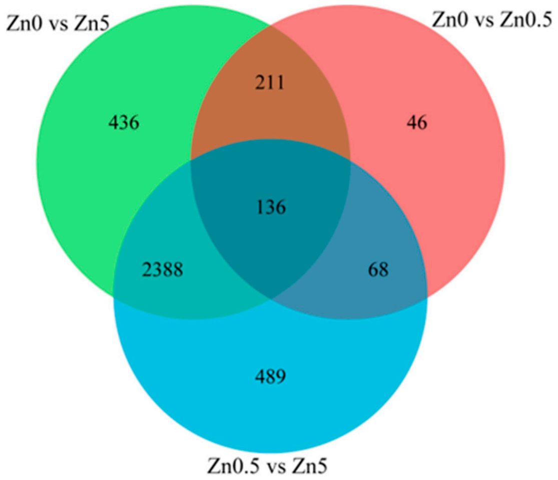

Figure S1. Venn diagram of DEGs in diverse comparison groups.

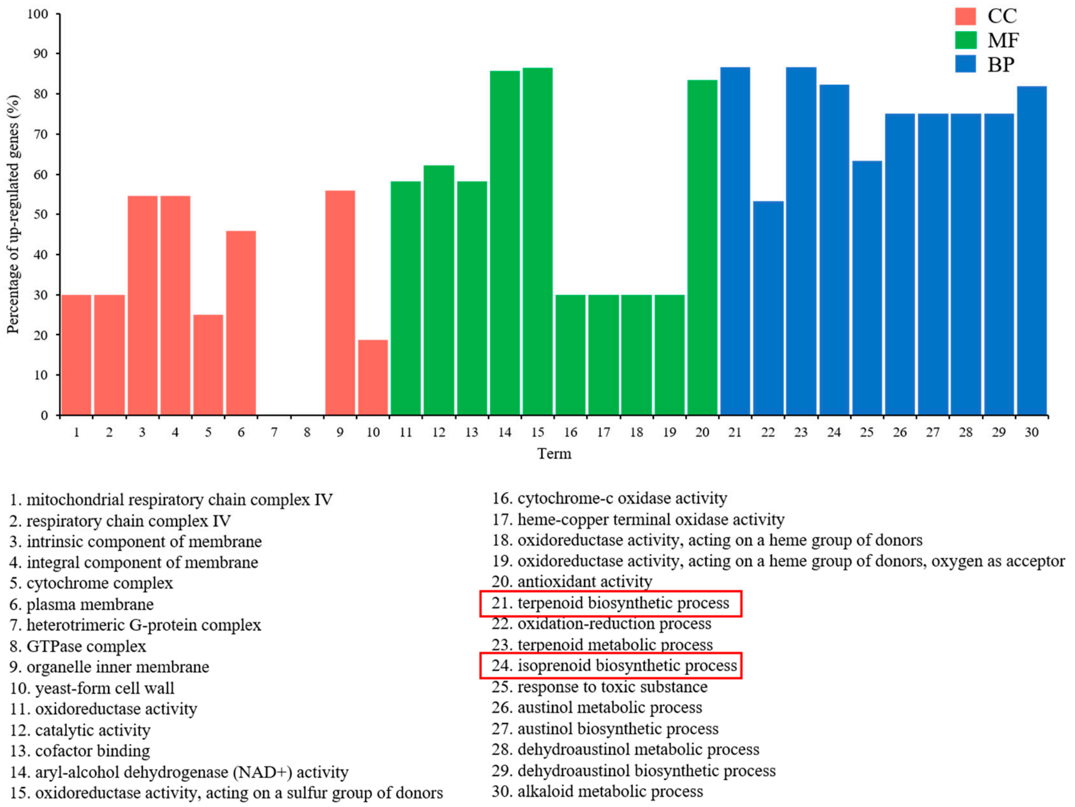

Figure S2. The percentage of upregulated DEGs to total DEGs in GO analysis comparing Zn5 and Zn0 groups.

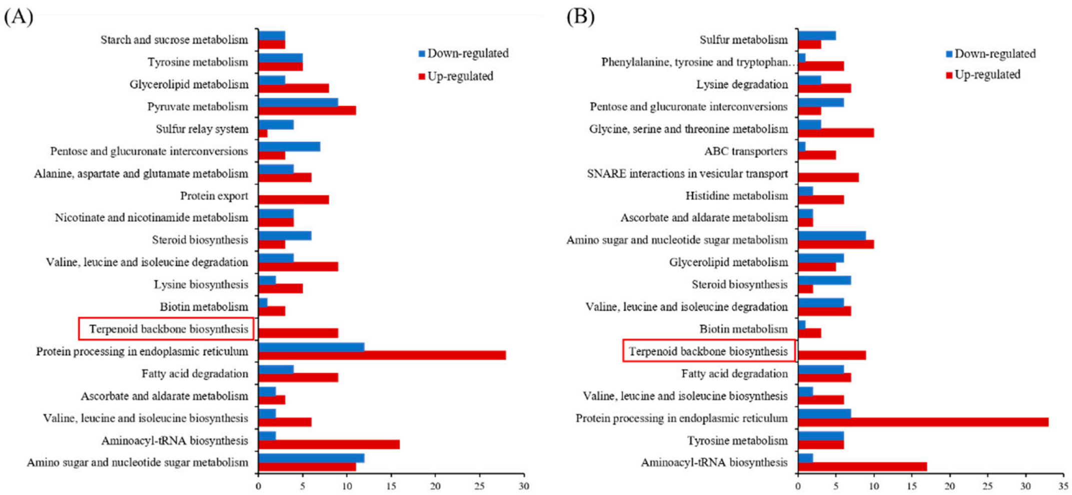

Figure S3. KEGG enrichment analysis of up- and downregulated genes in DEGs from diverse comparison groups. (A) Up- and downregulated genes in DEGs between Zn0 and Zn5 groups; (B) Up- and downregulated genes in DEGs between Zn0.5 and Zn5 groups.

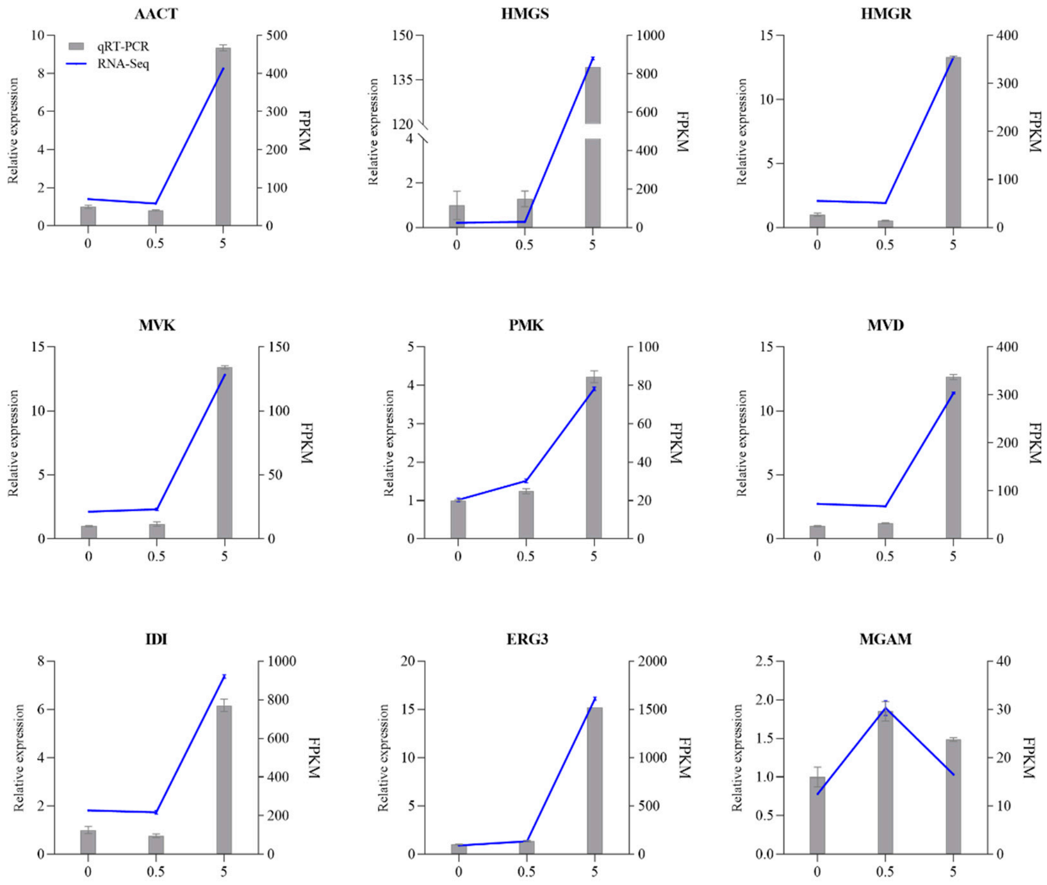

Figure S4. qRT-PCR validation of nine selected DEGs identified by RNA-Seq.

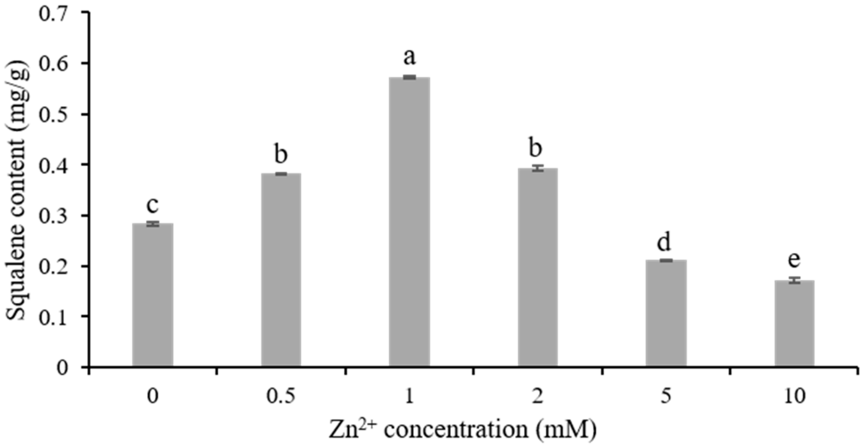

Figure S5. Squalene content of Sc-NTC induced by different Zn^{2+}concentrations. Error bars represent the standard deviation (SD) of three replicates ($n = 3$). Letters indicate a significant difference ($p < 0.05$).

Tables:

Table S1. transcriptome data statistics.

Sample	Clean Reads No.	Reads No.	Clean Reads (%)	Q30 (%)
Zn0_1	42122170	42642556	98.78	96.39
Zn0_2	42967442	43600618	98.55	96.07
Zn0_3	45263134	45811882	98.80	96.37
Zn0.5_1	38077878	38647518	98.53	96.13
Zn0.5_2	44843824	45573524	98.40	95.87
Zn0.5_2	41561084	42219924	98.44	95.98
Zn5_1	50091440	50825008	98.56	96.19
Zn5_2	47614472	48354366	98.47	96.02
Zn5_3	44733082	45393732	98.54	96.12

Table S2. Information about primers used in the present study.

Primers	Sequences (5´→3´)	Annotation
AACT-F	TCGGTGTCAAACCACTCGCAAAAAT	qRT-PCR
AACT-R	CCAGAGAAACGATAATACGGCAACC	
HMGS-F	CGAGAAGACTGCGAGGCGATAC	qRT-PCR
HMGS-R	TGGGACTTGAGGGATTGGAGAGG	
HMGR-F	TGGCTTTGAACGCCGTCTTA	qRT-PCR
HMGR-R	CTCTCCATTTCGTCGCTATCACC	
MVK-F	TTTGGAAAGAAAAGCGGAATGGAT	qRT-PCR
MVK-R	TTGCTTCCAACAGTCCTTCAACAT	
PMK-F	AAGATTGAGCCATTCCAACTCCCAC	qRT-PCR
PMK-R	GCAGCCTTATGCCACTTCAAAACTT	
MVD-F	TTTCGTATTTCCCGCAAGCCCAG	qRT-PCR
MVD-R	GCATTCAGCAAAGCCTCCTCAGC	
IDI-F	GCTGCTGCGGCGTTTGCTACTAT	qRT-PCR
IDI-R	ATCTTCTCGGATGCTCGTTGCTG	
ERG3-F	TCAAGTCCGCCAAGAGATTTACAT	qRT-PCR
ERG3-R	GGTGAAAAGAAGGAACCATAAGAT	
MGAM-F	TGGTGGACGGAGGCATTTAGGA	qRT-PCR
MGAM-R	GGATGAACGGCGTGGAAGTATT	
α-tubulin-F	CCAGCAAGCGTTACCGATT	qRT-PCR
α-tubulin-R	TCCACGACGTCCATCGTTC	
pYE-HMGS-F	AGGGAATATTAAGCTTATGGTTGCTGCGCCCAGTTC	seamless cloning
pYE-HMGS-R	CCCCCATGGTAAGCTTTCATGCTATCGGAGCACGGC	
T7F	TAATACGACTCACTATAGGG	colony PCR
C1R	GTGACATAACTAATTACATGATG	

[1] The underline represents the homologous arm sequence.
